# Facilitating factors and barriers to malaria research utilization for policy development in Malawi

**DOI:** 10.1186/s12936-016-1547-4

**Published:** 2016-10-19

**Authors:** Chikondi A. Mwendera, Christiaan de Jager, Herbert Longwe, Kamija Phiri, Charles Hongoro, Clifford M. Mutero

**Affiliations:** 1 Institute for Sustainable Malaria Control (UP ISMC), School of Health Systems and Public Health, University of Pretoria, Pretoria, South Africa; 2 Mailman School of Public Health, ICAP at Columbia University, Pretoria, South Africa; 3 School of Public Health and Family Medicine, College of Medicine, University of Malawi, Blantyre, Malawi; 4 Department of Population Health, Health Systems and Innovation, Human Sciences Research Council (HSRC), Blantyre, Malawi; 5 International Centre of Insect Physiology and Ecology (ICIPE), P.O. Box 30772, Nairobi, Kenya

**Keywords:** Facilitating factors, Barriers, Malaria research, Policy development, Research utilization, Malawi

## Abstract

**Background:**

Research on various determinants of health is key in providing evidence for policy development, thereby leading to successful interventions. Utilization of research is an intricate process requiring an understanding of contextual factors. The study was conducted to assess enhancing factors and barriers of research utilization for malaria policy development in Malawi.

**Methods:**

Qualitative research approach was used through in-depth interviews with 39 key informants that included malaria researchers, policy makers, programme managers, and key stakeholders. Purposive sampling and snowballing techniques were used in identifying key informants. Interview transcripts were entered in QSR Nvivo 11 software for coding and analysis.

**Results:**

Respondents identified global efforts as key in advancing knowledge translation, while local political will has been conducive for research utilization. Other factors were availability of research, availability of diverse local researchers and stakeholders supporting knowledge translation. While barriers included: lack of platforms for researcher-public engagement, politics, researchers’ lack of communication skills, lack of research collaborations, funder driven research, unknown World Health Organization policy position, and the lack of a malaria research repository.

**Conclusion:**

Overall, the study identified facilitating factors to malaria research utilization for policy development in Malawi. These factors need to be systematically coordinated to address the identified barriers and improve on malaria research utilization in policy development. Malaria research can be key in the implementation of evidence-based interventions to reduce the malaria burden and assist in the paradigm shift from malaria control to elimination in Malawi.

## Background

Developing countries, in view of their limited resources, need to take advantage of knowledge translation (KT) initiatives to maximize the utilization of research for health towards implementing interventions with proven track record [1]. It is evident that creative strategies, such as interaction between researchers and policy makers, are needed to promote utilization of research for policy making since traditional dissemination efforts have not yielded much change [2]. Research-informed policies have led to development of health interventions with improved health outcomes, ultimately saving lives [3]. It has also been recognized that research is critical in strengthening health systems and improving equitable distribution of scarce resources in low and medium income countries (LMICs) [4]. Such recognition should be the basis for supporting and utilizing research for the improvement of health systems. However, the effective use of such research remains a challenge in many LMICs where weak health systems exist and poverty-related disease burden remains high [5].

Despite a decrease of malaria prevalence among children age 6–59 months in Malawi from 43 % in 2010 to 33 % in 2014, malaria continues to be a major public health problem [6]. It is estimated that four million cases of the disease occur annually, mostly affecting children under the age of five years and pregnant women [6]. The Ministry of Health (MOH) through the National Malaria Control Programme (NMCP) has strived to implement the National Malaria Strategic Plan for 2011–2016 with the vision to reduce the malaria burden for all people in Malawi and attain a ‘Malaria-free Malawi’ through the scaling-up of malaria interventions [6]. It is, therefore, through implementation of evidence-based interventions that Malawi can reduce the malaria burden and shift from the paradigm of malaria control to elimination [7]. The adoption of malaria research utilization in policy development needs a systematic approach. Thus, a framework to facilitate this process needs to be developed. The overall objective of this study is to contribute towards the development of such a framework in Malawi.

### Conceptual framework

The majority of research-to-policy frameworks developed for research utilization are generally in the context of developed countries [8, 9]. Their applicability and relevance pose a challenge in LMICs [10]. It is essential that contextual factors are considered when developing such frameworks [11].

**Fig. 1:**
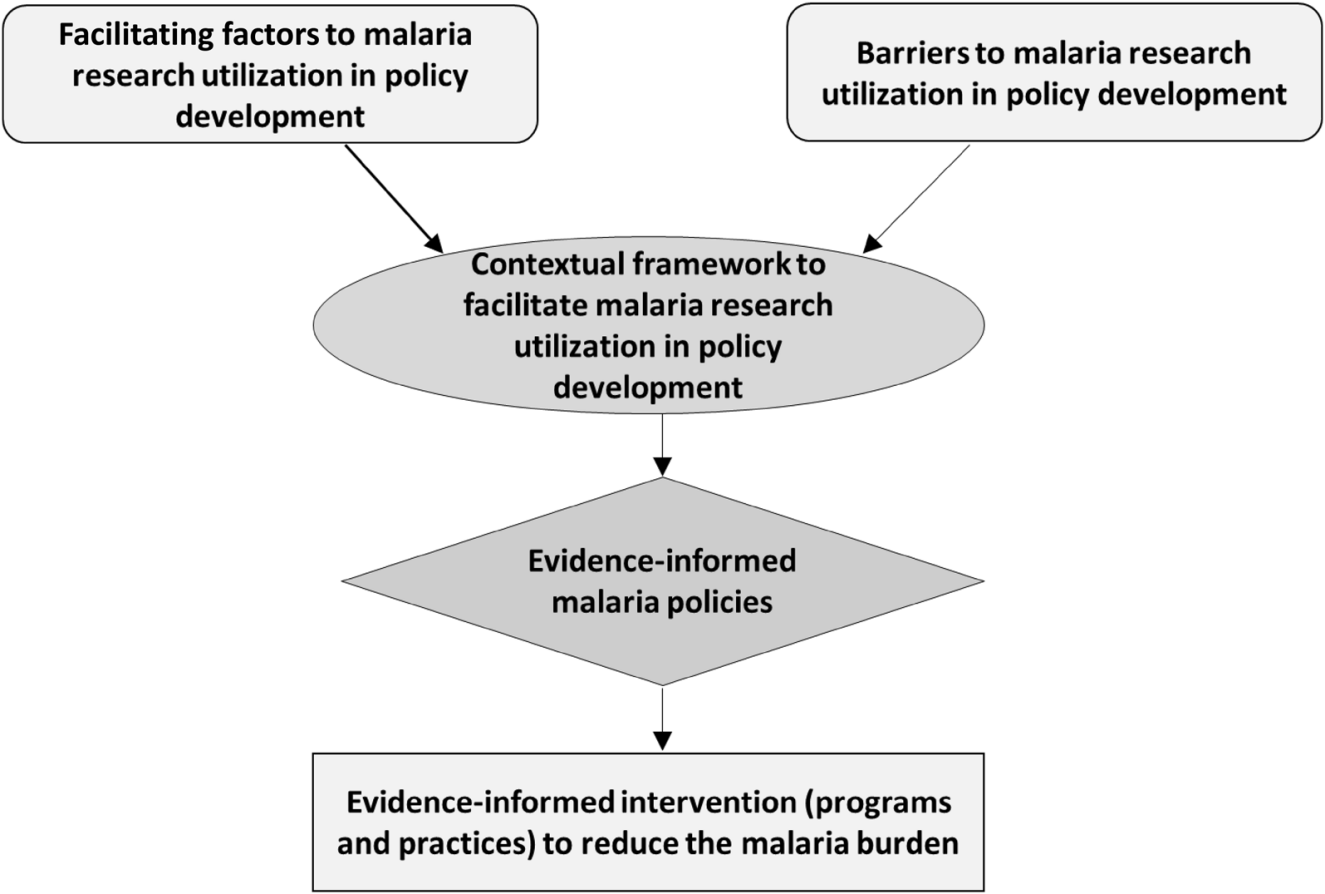
Study conceptual framework

The Ottawa Model of Research Use (OMRU), developed by Logan and Graham [9], guides the development of KT strategies for the improvement of health service in developing countries. Its basic principles require the assessment of enablers and barriers in the utilization of research for policy development in a particular system in order to develop a contextually relevant framework. This study’s conceptual framework (Fig. 1) was underpinned by this model. The specific objective of this study was to assess the facilitating factors and barriers to malaria research utilization for policy development in Malawi.

## Methods

The study applied qualitative research methods using in-depth interviews with key informants (KIs) conducted between April and July 2015. The sample population comprised individuals from malaria-related professionals, government officials and relevant stakeholders (Table 1). Purposive and the snowballing sampling techniques were used in identifying KIs. The interviews were conducted by the principal investigator. Data were captured using digital audio recorders in English, followed by transcription and importation of transcripts into QSR Nvivo 11, a software package for coding, organizing, management, and analysis. The analysis was based on the Grounded Theory principles in which data are thematically categorized and grouped into common themes and later examined contextually to explain the arising issues [12]. Verbatim quotes were used to illustrate concepts or points of view.

**Table 1:** Details of key informants (KIs)

KI	Gender	Current position and Institution	Experience	Role
1	Female	Malaria epidemiologist, Malawi—Liverpool Wellcome Trust (MLW)	Over 10 years in malaria research	Researcher
2	Female	Director, Blantyre Malaria Project	Over 20 years in malaria research	Researcher
3	Male	Director, Centre for Social Research	Over 20 years in malaria research	Researcher
4	Male	Medical epidemiologist, Director University of Northern Carolina Project	Over 20 years in malaria research	Researcher
5	Male	Biostatistician, National Malaria Control Programme (NMCP)	Over 5 years in malaria research	Researcher
6	Male	Epidemiologist, MLW	Over 10 years in malaria research	Researcher
7	Female	Public health specialist, MLW	Over 5 years in malaria research	Researcher
8	Male	Public health specialist, Malaria Alert Centre (MAC)	Over 5 years in malaria research	Researcher
9	Female	Senior Nurse, Kamuzu College of Nursing	Over 10 years in malaria research	Researcher
10	Male	Medical biologist, Chancellor College	Over 10 years in malaria research	Researcher
11	Male	Pharmacologist, College of Medicine (COM)	Over 10 years in malaria research	Researcher
12	Male	Malaria epidemiologist, COM	Over 10 years in malaria research	Researcher
13	Male	Medical epidemiologist—Director of Malaria Alert Center (MAC), COM	Over 10 years in malaria research	Researcher
14	Male	Senior scientist, MLW	Over 40 years in malaria research	Researcher
15	Female	Retired pediatrician and director of MAC, COM	Over 30 years in malaria research	Researcher
16	Male	Pediatrician, Ministry of Health	Over 30 years in malaria research	Researcher
17	Male	Pediatrician, Ministry of Health	Over 40 years malaria research	Researcher
18	Male	Clinical trialist	Over 10 years in malaria research	Researcher
19	Male	Entomologist, MAC, College of Medicine	Over 10 years in malaria research	Researcher
20	Female	Clinical epidemiologist, NMCP	6 years	Programme manager
21	Male	Disease control officer, NMCP	5 years	Programme manager
22	Male	Entomologist, NMCP	11 years	Programme manager
23	Male	Environmental health officer, NMCP	6 years	Programme manager
24	Male	Deputy director of planning, Ministry of Health (MOH)	3 years	Policy maker
25	Male	Director of Research, MOH	8 years	Policy maker
26	Male	Health economist, MOH	11 years	Policy maker
27	Female	Health planner, MOH	12 years	Policy maker
28	Male	Research and knowledge translation manager (MOH)	2 years	Policy maker
29	Male	Head of Sector Wide Approach, MOH	6 years	Policy maker
30	Male	Health planner, Director of Planning and Policy Development MOH	2 years	Policy maker
31	Male	Chief of Health Services, MOH	5 years	Policy maker
32	Male	Malaria advisor, World Health Organization	15 years	Stakeholder
33	Male	Health economist, director Abt Associates Inc	4 years	Stakeholder
34	Male	Global fund coordinator, MOH	2 years	Stakeholder
35	Male	Malaria resident advisor, US Centre for Disease Control	3 years	Stakeholder
36	Female	Policy and advocacy coordinator, African Institute for Development Policy	2 years	Stakeholder
37	Male	Policy development and analysis, Abt Associates Inc	4 years	Stakeholder
38	Male	Chief research service officer, National Commission for Science and Technology	16 years	Stakeholder
39	Male	Malaria program specialist	5 years	Stakeholder

Ethical clearance was obtained from the National Health Sciences Research Committee (NHSRC) in Malawi and the Faculty of Health Sciences Research Ethics Committee at the University of Pretoria (Ref No. 146/2013).

## Results

A total of 39 KIs were interviewed and the categories of participants included: 19 malaria researchers (from College of Medicine, Malaria Alert Centre (MAC), Blantyre Malaria Project (BMP), Malawi-Liverpool Wellcome (MLW) Trust, Centre for Social Research (CSR), University of Northern Carolina (UNC), and other renowned researchers working elsewhere); eight policy makers from the MOH; four programme managers from the NMCP; and eight stakeholders (from the World Health Organization (WHO), African Institute for Development Policy (AFIDEP), Global Fund, United States Aid and Development Fund (USAID), National Commission for Sciences and Technology (NCST), and Abt Associates Inc). Table 1 presents the experience and current positions of the KIs.

The interviews focussed on identifying facilitating factors for malaria research utilization in policy development, and barriers hindering this process. Table 2 summarizes the identified factors categorized into institutional, personal, and research-based as declared by research participants.

**Table 2:** Facilitating factors and barriers to malaria research utilization for policy development

	Specific factors
Facilitating research uptake factors	
Institutional factors	Global influence on the emphasis of evidence driven policies
	The establishment of the department of Research in Ministry of Health
	The revival of the Policy Development Unit (PDU) and the development of guidelines for policy development and analysis
	The availability of the National Health Research Agenda (NHRA)
	Availability of funding organization such as NCST, USPMI, and Global Fund
	Establishment of the Knowledge Translation Platform (KTP) and the Knowledge Translation Unit (KTU)
	Establishment of the African Institute for Development Policy (AFIDEP) in Malawi
	Technical Working Groups to discuss research, and annual research dissemination conferences
	Collaboration of the NMCP and research centres such as the Malaria Alert Centre (MAC)
Personal factors	Local Researchers trained in various malaria research disciplines
	Growing number of researchers interested in knowledge translation
Research factors	Availability of malaria research conducted in Malawi
	Availability of the NHRA that includes the malaria research agenda
	A position already established by WHO
Barriers to research uptake	
Institutional factors	Lack of a platform on which researchers can engage with the public
	Lack of research capacity at the NMCP
	Politics
Personal factors–researchers	Lack of research communication skills to policy makers
	Focus on publishing and career advancement
	Lack of collaboration of researchers with policy makers
	Lack of collaboration among researchers of different disciplines
Personal factors–policy makers	Lack of time to find and read research articles
	Inability of research synthesis
	Lack of motivation and rigid to change
	Mistrust of research findings
Research factors	Research not addressing the country’s needs
	Funder driven research
	Unknown WHO policy position
	Access to malaria research and lack of a malaria research repository

### Facilitating factors to malaria research utilization for policy development

#### Institutional factors

##### Global influence

Respondents acknowledged global efforts in promoting KT and were increasingly aware of the importance of research evidence in policy development. This is supported below:

*“The current movement worldwide is that research findings must find their way to policy and practice through systematic processes which are now undergoing by the title ‘knowledge translation platform’”.* (Policy maker)

This global effort has compelled developing countries, such as Malawi, to strive towards policies that are research driven.

#### Government efforts

##### Ministry of Health

Political will from government through the Ministry of Health was identified to be key in promoting research for policy development in the country. The government established the department of research in Ministry of Health with the aim of driving evidence-driven policies, while in 2012 it revived the Policy Development Unit (PDU) [13]. This was described below:

*“We had to revitalize from a scratch from 2012 what used to be the policy development unit to make it functional and be able to play a coordinating role to the healthy policies in the healthy sector in Malawi…and am happy to report that now the unit is coordinating various health policies”.* (Stakeholder)

To support its function, the PDU developed guidelines that provide a framework for harmonizing policy development and analysis in the public health sector [13]. Steps two, five and six in the guidelines require research evidence for decision-making. In its support the MOH developed the National Health Research Agenda (NHRA), which identifies priority research areas. As Malawi seeks to implement the NHRA, it is imperative to provide research funding. Government declared commitment through the allocation of at least 2 % of the District Implementation Plan (DIP) budget to research [14, 15]. However, this directive is not upheld due to constrained funding which results in research not being prioritized. It is against this funding gap that the NCST was established to provide grants to local researchers addressing the NHRA. The task of the NCST was alluded to by a stakeholder:

*“The national commission for science and technology is key in terms of contributing towards promoting the undertaking of research to contribute towards development of policy in the identified priority areas of research”.* (Stakeholder)

While other factors such as international institutions including the Global Fund and the United States’ President for Malaria (USPMI) as identified by respondents were committed to assist government in supporting malaria research.

The establishment of the Knowledge Translation Platform (KTP) in the MOH with assistance from Dignitas International was also identified by respondents as a government commitment to enhance research utilization in decision-making. The aim is to communicate research findings to policy makers addressing the challenge of many researchers, as highlighted below:

*“How do I make sure I translate my findings in an appropriate way to policy makers…how do we keep on highlighting our individual study findings as something that policy makers can take a decision on…so I think the separate developing groups of people who focus on knowledge translation can really use best practices”. (*Malaria researcher)

##### NMCP

Respondents recognized that the NMCP makes specific government efforts to facilitate malaria research utilization. The setting up of Technical Working Groups (TWGs) enables informal evidence sharing and debate. This was described below:

*“I like technical working groups because they are in a way rather informal where you can actually engage with the ministry of health officials and as researchers we can learn the key issues that the ministry is looking for”.* (Malaria researcher)

In addition to TWGs, the NMCP organizes the annual national malaria research dissemination conferences where malaria research supported by government are disseminated.

Respondents also highlighted as vital the collaborations of the NMCP and research centres. The lack of research capacity at the NMCP has compelled the programme to rely on research institutions to conduct policy-relevant research on its behalf. NMCP will engage a particular research centre based on the type of study required. For example, a strong collaboration exists between NMCP and MAC, which conducts operational research providing evidence that directly feeds into policy. This was described below:

*“Malaria Alert Centre is directly involved with the NMCP from the inception of a study, so it’s not hard when we are presenting the results for policy changes because they are involved”.* (Malaria researcher)

Other important research institutions that provide evidence to the NMCP include; MLW trust that builds capacity and conducts high quality clinical research in the country. The efforts of this institution supplement those for the government because it is externally funded by the Wellcome Trust shouldering the challenge of funding faced by the government. Therefore, the research institution is able to conduct research in collaboration with the NMCP for policy development in addition to exploratory or basic research, CSR which conducts social-cultural research, the Blantyre Malaria Project (BMP) focusing on severe malaria research, the UNC conducting clinical research.

##### University of Malawi, College of Medicine efforts

College of Medicine is one of the five constituent colleges in Malawi conducting health research. The establishment of the Evidence Informed Decision-making Centre (EvIDenCe) in 2015 at the institution was identified as key and a reflection on the commitment to enhance translation of research into policy and in conducting policy relevant research. EvIDenCe was established to drive KT efforts at the college including; conducting and teaching systematic reviews, evidence synthesis and writing policy briefs for policy decisions, teaching evidence-based health care, and contributing to the formulation of the NHRA. Similarly the College of Medicine conducts annual research dissemination conferences where malaria research results are also disseminated. In supporting the NMCP malaria conference, these conferences provide a platform for policymakers to be aware of malaria research conducted in the country. Challenges emerge when government officials partially attend such conferences and respondents felt that further efforts such as the TWGs by the NMCP should be made to engage with them. Separate interactions with policymakers, which highlight research implications on policy, need to be organized. Research findings should include their policy implications.

#### AFIDEP efforts

To complement the work by KTP and EvIDenCe, respondents identified the AFIDEP that was established in Malawi in 2013. AFIDEP focuses its work in knowledge utilization, capacity strengthening, and knowledge synthesis and translation. Through the Strengthening Capacity to Use Research Evidence in Health Policy (SECURE) programme, AFIDEP strengthens the capacity of health policy makers and legislators in research evidence utilization for decision making. Their aim is to consolidate interaction of researchers and policy makers and hence improve on the trust for each other. One of its specific activities of interest is building the capacity of policy makers to access, appraise and apply research evidence in their decision making and policy development. This initiative is vital in instilling a culture of evidence use for decision-making among policy makers and its efforts were recognized by a stakeholder as below:

*“The initiative of some programmes like AFIDEP are very good because those can help to mobilize resources for local initiatives”.* (Stakeholder)

#### Personal factors

Respondent acknowledged that it was key for knowledge creation since local researchers are being trained in various research disciplines, such as malaria in pregnancy, immunology, parasitology, human immunodeficiency virus (HIV) and malaria, cerebral malaria, and malaria treatment. In addition, some researchers are focusing on knowledge translation and health systems research. The quote below supports this:

*“I think there is a real momentum or opportunity now with a building of capacity of people with interest in knowledge translation, in health systems research and in policy process…we have people who know better how to review the current status of research and internationally link to what is available on the national level and identify the gaps but they also know how to develop policy briefs, which I as a researcher don’t have that much experience”.* (Malaria researcher)

#### Research factors

The most important element in facilitating research evidence for policy development is availability of local research findings. Respondents acknowledged that ample malaria research is being conducted in Malawi. This is described below:

*“I mean too many studies have been done in malaria, sharing of these research results I think sometimes is not there”.* (Malaria researcher)

Although the amount of available research is critical, the quality of research is equally important for policy development. As indicated above the collaboration between the NMCP and MAC was identified as prominent. MAC is a recognized research program called International Centres of Excellence for Malaria Research (ICEMR) that conducts high quality research with the purpose of addressing the malaria burden locally and internationally. The aim is to develop evidence-based interventions for the Country and hence, research conducted at MAC has direct bearing on policy. Therefore, policy makers have confidence in the evidence they provide. This was highlighted as below:

*“Being an ICEMR means that we have what we call a powerful front that when we produce the results they have already been reviewed by all these bodies and when we make recommendation to the ministry it is really powerful because even the ministry knows that it’s coming from a very powerful front and it has been reviewed by collaborators internationally”.* (Malaria researcher)

The research conducted is guided by the NHRA, hence there is availability of research that is specifically responding to needs of the country.

They (respondents) also admitted that the research conducted in most of the time in line with WHO recommendations hence it is often used symbolically in policy development.

### Barriers to malaria research utilization for policy development

#### Institutional barriers

One of the major barriers to research utilization identified was the lack of a direct link between researchers and critical societies of policymaking other than the MOH. These societies include the Civil Society Organizations (CSOs), influential people such as chiefs, and the public. These sentiments were expressed as below:

*“If you are going to really influence policy you shouldn’t only be influencing people within the Ministry of Health, but the people for whom policy is being made for, without that platform you don’t have community based organizations, [Non*-*Governmental Organisations] NGOs or even traditional chiefs, and influential people in the community actually contributing to the policy makeup”.* (Malaria researcher)

Respondents felt that such a platform, as in many developing countries, can have advocacy through newspaper campaigns, television, radio, and other social media channels.

Another identified barrier was the limited research capacity of NMCP, which has often delayed adoption of research findings. The NMCP has on several occasions relied on independent researchers or research institutions. Consequently, the NMCP would rather wait for WHO recommendations, which may come late, while local evidence has shown the need to develop new innovations. This was observed as below:

*“The NMCP will wait for WHO because they don’t have the capacity sometimes to actually evaluate the evidence, now for WHO to have a stamp on it you have to carry out quite extensive studies and there is a lag time and you lose out on possible benefits of a strategy that is more locally effective”.* (Malaria researcher)

Participants recognized political impact on policy development. Research can provide good evidence and recommendations but politicians can have preconceived policy positions contrary to the evidence, which leads to researchers’ frustration. This was referred by a researcher:

*“For people to appreciate researchers they have to have an interest in implementing data driven policies, so they will always absorb research if they have that kind of interest, sometimes you may get in a situation where you might be discussing certain things but people have preconceived ideas already about what they want the situation to be even if the data may be speaking otherwise”.* (Malaria researcher)

#### Personal barriers

##### Researchers

It was revealed that researchers find it challenging to effectively communicate their findings to policymakers. Somehow researchers assume that their work ends once they publish or present their results and it is the responsibility of the policymakers to make use of the findings. A researcher made the narration below to confirm this:

*“My role ends when I present the results…if the Ministry wants they can make a decision on how they are going to utilize those results to develop policies. As far as I am concerned as a researcher I will do the research and make a presentation…that’s all I know but the question is do I have the skills to translate whatever the results I have into something that the Ministry of Health can easily utilize…I think most of us as researchers don’t have that experience of translating the research results into something that the ministry of health can easily use”.* (Malaria researcher)

Respondents expressed gratitude with the timely establishment of KTP and EvIDenCe which can tackle these challenges by focusing on bridging the gap between researchers and policymakers.

Another barrier highlighted how some researchers are naïve in the policymaking process and assume that only their findings can influence policy. In addition, they may not collaborate with the NMCP or policymakers in the research process. The NMCP acknowledged this:

*“But one very critical thing is collaboration, because sometimes people do studies, like check for insecticide resistance and tell us that there is resistance in the whole country…we have no idea and [we] don’t know the person and the protocol. That will not change the policy even if the results are good, it will not, because that was academic”.* (Programme manager)

In other cases the NMCP may not be aware of research findings, especially if such research was conducted by foreign researchers. These findings are disseminated externally and published in inaccessible journals, as lamented below:

*“Unfortunately some of these studies are not even known to the Ministry…people come to this country from out there to do their research in different parts of this country…it is only when you go to a conference abroad and you find there are a lot of papers on Malawi”.* (Malaria researcher)

Lack of collaboration among researchers was also mentioned to pose a challenge. In many situations researchers work in isolation by focusing in one area, such as drug or vaccine discovery, without engaging in other research disciplines. The need for collaboration among researchers is important in providing a variety of evidence that policymakers can use. A researcher acknowledged this:

*“We are usually working in narrow areas and we don’t think about other discipline that would actually strengthen our research work. So in the planning phase of all these research studies we should be looking for collaboration”.* (Malaria researcher)

##### Policymakers

Respondents indicated that most policymakers lack time and the ability to find research articles in journals and synthesize the evidence. They lack expertise in research exploration and interpretation of scientific findings. Journals may not be readily accessed and the evidence from different publications might be contradictory, thereby reducing the confidence policymakers have for a given set of research findings. A researcher testified to this:

*“Most of the policymakers, do not have time to go through journals and read what research is. They say it but [actually] in actual sense they don’t read and even if you read, you will get two [to] three articles [you can get articles] that are saying different things”.* (Malaria researcher)

Lack of trust in the findings by policymakers may also arise when they do not understand the research process. They may have problems with generalization of findings. This was supported by the quote below:

*“There are always issues [of] about generalization…but sir you did this in Chikwawa, how do we know that it applies to the whole country?”* (Malaria researcher)

Respondents indicated that collaboration between researchers and policymakers can address such problems as they get oriented to the research process. It was also revealed that in some cases policymakers lack motivation and can be reluctant to change. This is common among personnel who are used to operating on older policies and routine activities. Such personnel are reluctant to adopt current evidence for policy change.

#### Research barriers

Respondents felt research that does not address the country’s needs is difficult to be utilized. Partly this problem comes when research funding is provided by a donor with their own research focus, but respondents felt that it should also be the responsibility of the government, if it intends to utilize such findings, to provide funding that can generate the required evidence. These sentiments are expressed below:

*“If Malawi really wants to answer its own questions that are pertinent then Malawians have to come up with resources for research. And most of this research that we are talking about is not expensive research, this is research that can be conducted with very minimal resources”.* (Malaria researcher)

A further challenge was revealed when the WHO does not have an established policy position on the issue at hand. Policymakers become reluctant to make a move until WHO has a position. This was affirmed as follows:

*“Evidence normally translates quite slowly into a policy especially in sub*-*Saharan Africa. Since we tend to depend a lot on guidance from international agencies. Where we have a feel about a particular thing that does not work really well for us, we still want to wait from some [the] prompting from these international bodies for us to move”.* (Malaria researcher)

A critical barrier mentioned to malaria utilization was the lack of a repository for local malaria research. Usually the research reports are scattered across research institutions or ethical approval bodies, which makes it difficult to access. Such sentiments are shared below:

*“The problem is limited access to data, people do research but I don’t think you have a platform where you can put your report, like a single repository of malaria research in Malawi where if I want something I’ll just click and get a link or get somewhere where I can get malaria specific research. So without that kind of a repository of information for malaria, for us as a country decision makers are often faced with a challenge of knowledge gap when we actually have enough information”.* (Stakeholder)

## Discussion

The notion of increasing utilization of research evidence in policy formulation has gained global level focus [16]. However, putting in place structured efforts to support KT is critical for its achievement [1]. One of the initial steps in developing such structures involves the assessment of contextual factors for research utilization in a system in order to pragmatically address the barriers while maximizing on the supportive factors. This study assessed the various factors with the aim of contributing to the utilization of malaria research in policy formulation for reduction of malaria burden in Malawi.

Global efforts were identified to be key in facilitating research utilization in policy development. For instance the Evidence to Policy Network (EVIPNet) [17], an initiative by WHO, has encouraged African countries to establish KTPs [18]. These KTPs aim at bridging the gap between researchers and policymakers by creating an environment for interaction. This initiative compelled Malawi with assistance from Dignitas International to establish its first KTP in 2012 [18], which has advocated and enabled an environment for research utilization in policy development [19]. Similarly, the College of Medicine, a research and academic institution created EvIDenCe, signifying efforts of translating research evidence into policy.

Political will is important for Malawi if it aims to adopt the malaria elimination paradigm [7]. This can be achieved through implementation of research-informed policies. This commitment has led the government to establish the department of research within the MOH and develop relevant tools to assist the development of research-informed policies. These tools include guidelines for policy development and analysis, and the NHRA. The guidelines offer fifteen important practical steps in policy development and these include; (1) Assessment of the legal and policy framework in order to prioritize areas in need of new or revised policies, (2) identification or analysis of problems and issues than need to be addressed in or revised policies, (3) organisation of the policy development process, (4) development of policy objectives for the envisioned policy, (5) identification of policy options to achieve the developed goals, (6) Evaluation of the policy options on their feasibility, (7) selection of the appropriate policy option, (8) Drafting the policy, (9) circulation of the draft policy to stakeholders for input and revision, (10) obtaining official policy endorsement from MOH senior management, (11) securing of any needed legal or regulatory changes and explore any lower-level policy documents for support and consistency, (12) launch and implement the policy, (13) monitor and evaluate the policy, (14) learn from the monitoring and evaluation, and (15) revise the policy as needed. Most important are steps two, five, and six, which seek evidence for decision-making [13]. Working closely in support of these guidelines is the NHRA, which identifies the country’s health research needs. As researchers respond to the NHRA they provide relevant local evidence that is conveniently available for policy decision-making.

It is thus imperative for government to support such research. However, amid challenges of government research funding, certain institutions such as the NCST offer research grants supporting studies responding to the NHRA. In addition, organizations such as the USPMI and the Global Fund pledge research support for policy development. These institutions aim to contribute to the shared vision of a malaria-free world by the Roll-Back Malaria partnerships and target for goal number three of the sustainable development goals [20–22].

The importance of interactions between researchers and NMCP or policymakers can never be over-emphasized. Through TWGs, researchers have acknowledged that they understand the needs of NMCP and policymakers while they also appreciate the research process. Other interactions occur during the annual research dissemination conferences. Such interactions enhance the uptake of research findings since policymakers are aware and can contribute to the research process [23, 24].

Another vital interaction occurs during collaboration of the NMCP and research institutions. Similar arrangements between the Ontario Drug Policy Research Network and the Ontario Public Drug Programme have revealed the importance of such a collaboration for research to be timely conducted and used for policy development [25]. Importance should also be placed on multidisciplinary research. With a growing diversity of malaria researchers in Malawi, multidisciplinary research can increase the utilization of research findings and attract funding since funders are inclined to support such research [24].

The barriers to malaria research utilization which the study found included the lack of a platform for researchers to engage with the public. Public opinion can be a strong force to influence policy change [1]. If the public grasp policy implications of research, they can be in a position to demand for better policies. It is strategic for researchers to engage with CSOs and the media in order to communicate research findings for purposes of influencing public opinion and advocate policy change [26, 27].

Politics can form a barrier to research utilization in the system. For instance, many senior positions in the MOH are political appointments which are subject to staff transfers. This can negatively impact policy processes as new personnel bring new ideas or lack the motivation to pursue previous efforts left by others. This is further exacerbated whenever there is a change of government [28]. Efforts should be made to retain personnel who initiate an activity until continuity is established.

Lack of research synthesis skills by policymakers has also been reported in other settings by Santesso et al. [1] who identified policymakers’ lack of skills and poor education background to apply and use research as common barriers in developed countries. This is amplified by the fact that researchers too lack the skills of communicating their research findings to policymakers. This study has identified the initiative by AFIDEP to strengthen the capacity of health policymakers and legislators in research evidence utilization for decision-making. This initiative is vital in instilling a culture of evidence use for decision-making among policymakers, which has been cited as one of the main barriers of knowledge translation [29].

Furthermore, the type of research conducted forms a pivotal role for its adoption into policy. This study revealed how research can be a barrier to its utilization. This was mainly through research that did not address the country’s needs simply because it was funder driven or it was for academic purposes.

Sometimes local evidence can show the need for policy change, but policymakers are reluctant if WHO has not yet made a policy position. This is a barrier as researchers become demotivated to provide evidence. However, if research is strongly siding with a WHO position it is likely to be used symbolically in supporting the policy position [30].

### Limitations of the study

All efforts were made to reach out and include all individuals who were key players in malaria research and malaria policy development. However, some prominent and experienced researchers and policymakers were either out of the country or had retired, rendering them unreachable. Their views could perhaps have provided additional perspectives to the study. However, to strengthen the study prominent individuals were interviewed in their current positions without new themes emerging. This indicated that a point of saturation was attained.

## Conclusion

The study has identified a number of facilitating factors and barriers that can enhance or derail the utilization of malaria research in Malawi. The identified facilitators and institutions offer hope of overcoming the barriers to malaria research utilization for policy development. It is important to have a systematic approach in coordinating these factors, and hence the need to develop a framework that can facilitate this process. The development of this framework is built on the identified institutions by creating links of collaborations based on the enhancing factors in order to tackle the barriers. Therefore, the framework will act as a guide to researchers, stakeholders, and policy makers to engage formally and utilize malaria research in policy development.

## Abbreviations

AFIDEP: African Institute for Development Policy
BMP: Blantyre Malaria Project
CSR: Centre for Social Research
CSOs: Civil Society Organizations
DIP: District Implementation Plan
EvIDenCe: Evidence Informed Decision-making Centre
EVIPNet: Evidence to Policy Network
HIV: Human Immunodeficiency Virus
KIs: Key Informants
KT: Knowledge Translation
KTP: Knowledge Translation Platform
LMICs: Low and Medium Income Countries
MAC: Malaria Alert Centre
MLW: Malawi-Liverpool Wellcome Trust
MOH: Ministry of Health
NCST: National Commission for Sciences and Technology
NHRA: National Health Research Agenda
NHSRC: National Health Sciences Research Committee
NGOs: Non-Governmental Organisations
NMCP: National Malaria Control Programme
OMRU: Ottawa Model of Research Use
PDU: Policy Development Unit
TWGs: Technical Working Groups
UNC: University of Northern Carolina
USAID: United States Aid and Development Fund
USPMI: United States President’s Malaria Initiative
WHO: World Health Organization
